# The relationship between Alzheimer disease and thyroiditis: A two-sample Mendelian randomization study

**DOI:** 10.1097/MD.0000000000035712

**Published:** 2023-11-03

**Authors:** Haiyang Yu, Xuejie Song

**Affiliations:** a Henan University of Chinese Medicine, Zhengzhou, Henan, China; b Henan Provincial Key Laboratory of Prescription-Syndrome Signal Transduction of Traditional Chinese Medicine, Zhengzhou, Henan, China; c International Joint Laboratory of Prescription-Syndrome Signal Transduction of Traditional Chinese Medicine in Henan Province, Zhengzhou, Henan, China.

**Keywords:** Alzheimer disease, causal relationship, Mendelian randomization, thyroiditis

## Abstract

This study aims to investigate the causal effect of Alzheimer disease on thyroiditis using medical English and the Nature journal style. Genome-wide association data for Alzheimer disease and thyroiditis were obtained from the Mendelian Randomization (MR) platform. Single nucleotide polymorphisms (SNPs) significantly associated with Alzheimer disease were identified and used as instrumental variables (IVs) to examine the causal relationship between Alzheimer disease and thyroiditis, employing a 2-sample MR study design. Five statistical methods, including inverse-variance weighted (IVW) method, weighted median estimation, simple mode estimation, weighted mode estimation, and MR-Egger regression, were utilized. In the study, 13 single nucleotide polymorphisms (SNPs) were identified to be significantly associated with Alzheimer disease (*P* < 5 × 10^–8^, linkage disequilibrium r^2^ < 0.001). Upon evaluation using different methods, a consistent association between Alzheimer disease and thyroiditis was observed inverse variance-weighted method [IVW]: odds ratio [OR] 1.32, 95% confidence interval [CI] 1.01–1.72; weighted median estimator: OR 1.32, 95% CI 1.01–1.72; Mendelian randomization Egger regression: OR 1.29, 95% CI 0.92–1.81), indicating a positive correlation between Alzheimer disease and increased risk of thyroiditis. There was no evidence suggesting that the observed causal relationship between Alzheimer disease and thyroiditis risk could be influenced by pleiotropy (Mendelian randomization Egger intercept 0.0058, *P* = .88. Our MR analysis reveals causal association of Alzheimer disease and thyroiditis, despite observational studies reporting an association between Alzheimer disease and thyroiditis.

## 1. Introduction

Alzheimer disease (AD) is a neurodegenerative disorder characterized by insidious onset and progressive development.^[1]^ Clinically, it manifests as memory loss, aphasia, disability, executive dysfunction, visual impairment, as well as personality and behavioral abnormalities.^[2,3]^ The prevalence of AD in the elderly population aged 65 and above is reported to be 3.21%.^[4]^ Thyroiditis (TH) is an organ-specific autoimmune disease mediated by various factors, often leading to abnormal fluctuations in thyroid function. Notably, several thyroid function parameters in the serum of AD patients exhibit significant differences compared to those in the general population, suggesting a potential association between AD and TH.^[5,6]^However, the causal relationship between AD and TH remains uncertain.

Observational studies are often confounded by reverse causation and confounding factors, resulting in insufficient evidence. Molecular-level randomized controlled trials are challenging to conduct due to ethical and methodological constraints. Mendelian randomization (MR) is an approach to infer causality between a given exposure and outcome based on Mendel law of independent assortment. It introduces the concept of instrumental variables (IVs), using genetic variation as a proxy for the exposure of interest, thereby simulating the association between exposure and outcome. MR effectively circumvents confounders and reverse causation found in traditional epidemiological study designs.^[7]^

In this study, we propose the use of genome-wide and epigenome-wide association study data combined with the Mendelian randomization (MR) method to explore the causal relationship between AD and TH.^[8,9]^ AD-related genetic variants will be employed as IVs, allowing us to infer the causal relationship between these 2 conditions.

## 2. Method

### 2.1. General information

This study aims to investigate the causal relationship between Alzheimer Disease (AD) and Thyroid Hormone (TH) by utilizing a 2-sample Mendelian randomization (MR) analysis method. AD serves as the exposure factor, with significantly Alzheimer Disease-associated single nucleotide polymorphisms (SNPs) used as IVs. The outcome variable is TH. An MR approach must fulfill 3 conditions: selected IVs must be associated with the exposure factor; genetic variability (IV) should not be associated with any confounding factors; and genetic variability (IV) must represent the occurrence of AD without being related to other alternative pathways.

### 2.2. Data sources

The AD data is derived from a genome cohort of 54,162 European ancestry volunteers (37,154 controls and 17,008 cases) with the ID number ieu-a-297. The TH data includes 214,694 European ancestry participants (213,693 controls and 956 cases) with the ID number finn-b-THYROIDITIS.

### 2.3. Statistical analysis

All statistical analyses were performed in R software 4.1.3, employing the TwoSampleMR package.^[10]^ The study utilized R software to screen all significant gene loci independently associated with AD as IVs. To select effective IVs and avoid interference from linkage disequilibrium effects, SNPs related to an increased risk factor for TH were excluded. The GWAS catalog was also searched to remove previously reported SNPs associated with complex traits.

#### 2.3.1. Verifying causal relationships.

The assessment of the causal effect of exposure on outcomes was analyzed using the inverse variance weighting (IVW) method. The IVW is akin to 2-stage least squares or allele score analysis crafted using individual-level data,^[11]^ and is adopted here as the principal MR analysis. The IVW method does not require individual-level data and can directly compute the causal effect values using summary data. In the absence of horizontal pleiotropy or when horizontal pleiotropy is balanced, IVW can yield unbiased estimates. To interpret directional pleiotropy and further validate causal effects, the results are compared with 4 other MR methods: the weighted median method,^[12]^ MR-Egger regression method,^[13]^ weighted mode-based simple estimate,^[14]^ and simple mode-based weighted estimate, thereby ensuring the stability and precision of the results.

#### 2.3.2. Sensitivity analysis.

MR-Egger regression intercept term tests were utilized to assess potential pleiotropy effects. A zero-intercept indicates no horizontal pleiotropy. Leave-one-out (LOO) analyses were further conducted to identify possible outliers. Each SNP was removed one at a time and combined effects were evaluated. Additionally, Cochran Q test was used to assess result heterogeneity, with a statistically significant *P* value indicating heterogeneous findings.

## 3. Results

### 3.1. Determining instrumental variables

In the AD dataset, a total of 16 SNPs were extracted as IVs, while only 15 SNPs (excluding rs9272561) were identified in the TH dataset. The harmonize data function was employed to maintain consistency in the effect allele direction and effect size for SNPs within both datasets (Supplementary Table 1, http://links.lww.com/MD/K460). SNPs rs12972156 and rs12977604, exhibiting intermediate allele frequencies, were subsequently removed.

### 3.2. Mendelian randomization results for two-sample analysis

**Figure 1.**
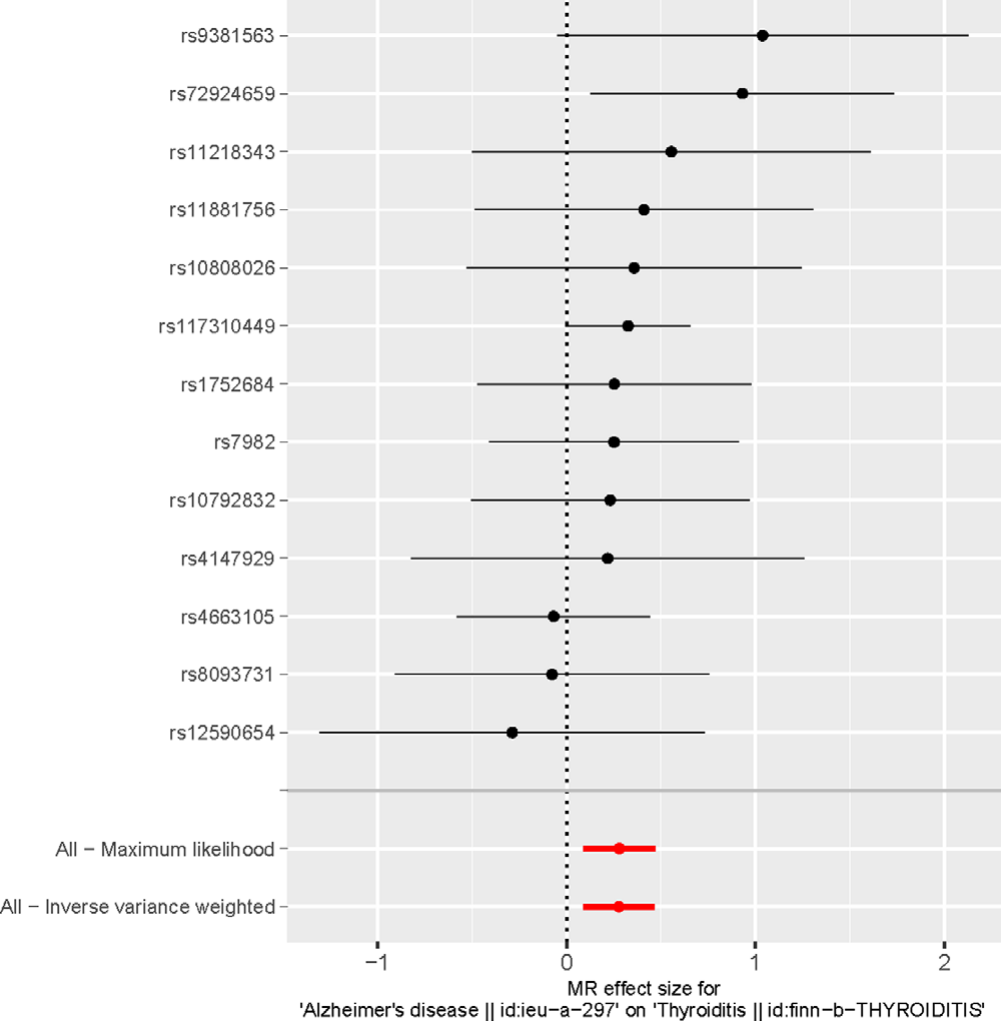
Forest map of SNP effect size. SNPs = single nucleotide polymorphisms.

**Figure 2.**
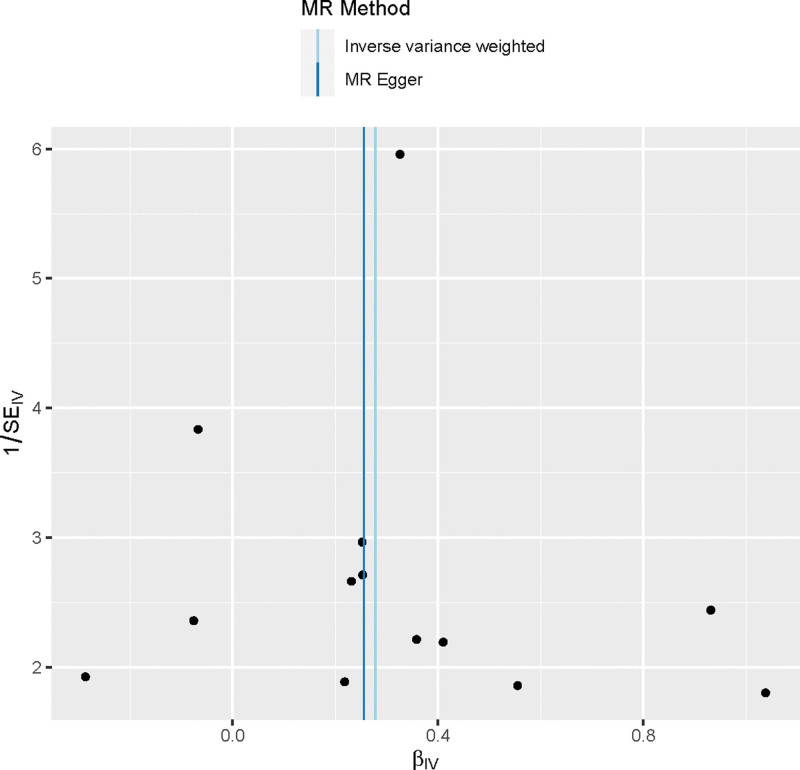
Mendel randomized funnel diagram.

Using the IVW method, we discovered that Alzheimer disease (AD) increases the risk of TH disorders (OR = 1.32, 95% CI: 1.09–1.59, *P* < .001). Consistent conclusions can be drawn through different analytical methods: Weighted median (OR = 1.32, 95% CI: 1.01–1.72, *P* < .05); MR-Egger (OR = 1.29, 95%CI: 0.92–1.81, *P* = .16) (Supplementary Table 2, http://links.lww.com/MD/K461). Forest plots depict the risk values mediated by individual SNPs for TH disorders (Fig. 1). Funnel plots confirm a symmetric distribution of causal effects obtained from each IV, suggesting that the results are less likely to be influenced by potential biases and possess good stability (Fig. 2).

### 3.3. Sensitivity analysis

**Figure 3.**
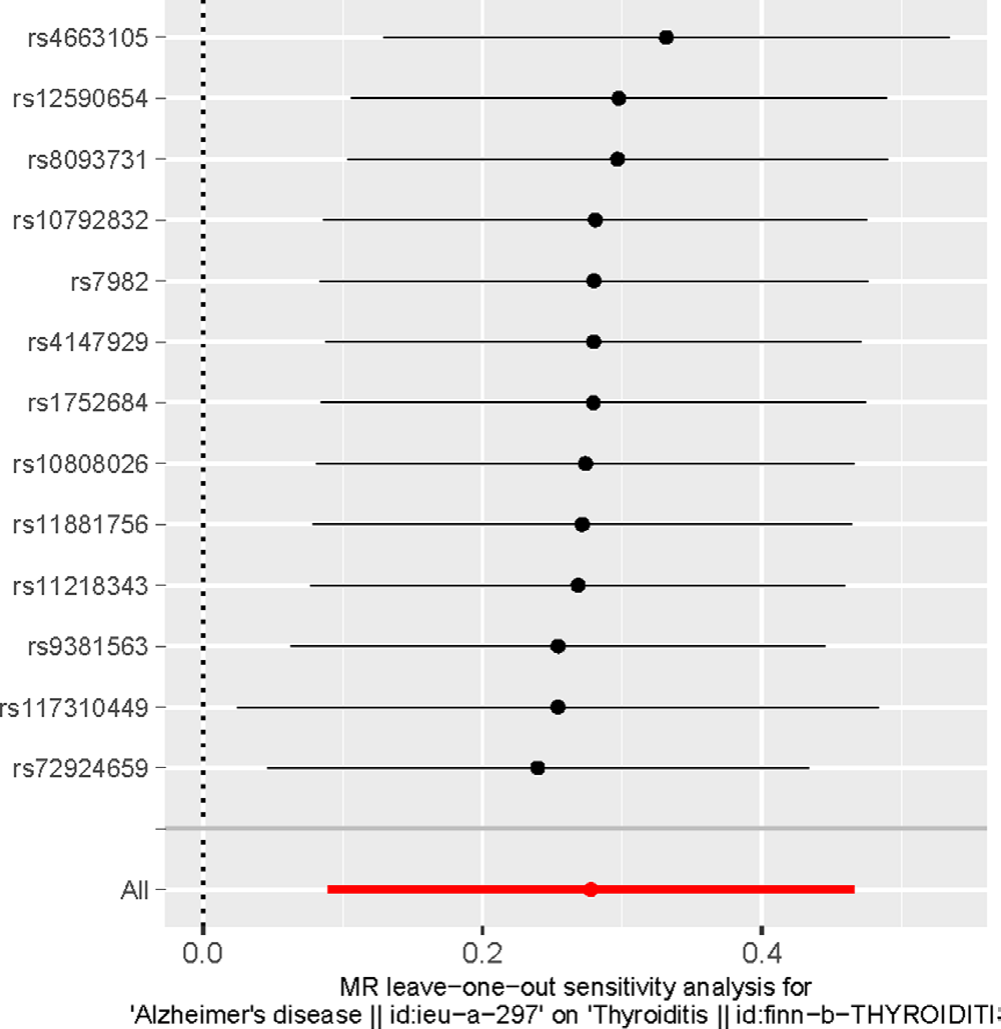
Leave one out sensitivity analysis results.

**Figure 4.**
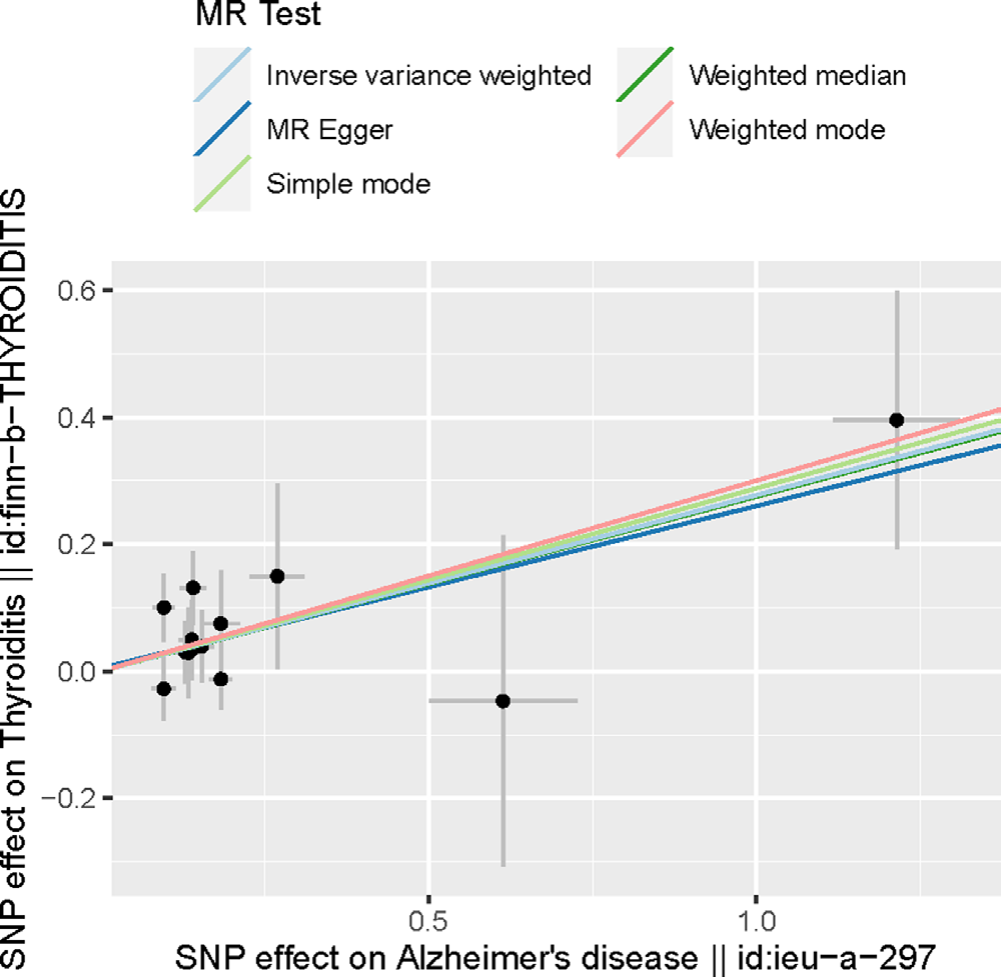
Mendel randomized scatter chart.

The intercept of the MR-Egger regression was 0.005 (*P* = .88), indicating that there was virtually no pleiotropy in the IVs. LOO analysis revealed no significant influence of individual IVs on the results, as the causal association remained statistically significant after excluding any single SNP. No strong effect of IVs on the outcomes was identified (Fig. 3). Cochran Q test result (Q = 8.55, *P* > .05) demonstrated the absence of heterogeneity. Furthermore, the LOO approach indicated no substantial difference between the combined effects of the remaining SNPs and the overall results when individual SNPs were sequentially removed. This finding further validated the reliability of the results. The scatterplot also corroborated the association credibility (Fig. 4).

## 4. Discussion

The majority of researchers believe that Alzheimer disease (AD) is the most common age-related progressive neurodegenerative disorder, linked to the thyroid system, dopaminergic neurotransmission, serotonergic system, and cholinergic system.^[14]^ It can cause significant social dysfunction, language communication barriers, and the loss of daily living abilities in elderly patients.^[15]^ In a cohort study of 50 AD patients, their hypothalamic-pituitary-thyroid axis was disrupted, and levels of serum thyrotropin-releasing hormone, TSH, total T4 (TT4), total T3 (TT3), FT4, and FT3 were significantly decreased compared with the control group.^[16]^ Subclinical hyperthyroidism elevates the risk of dementia and AD, particularly in elderly AD patients who are positive for thyroid peroxidase antibodies.^[17]^ Another study suggests that patients with subclinical hyperthyroidism and TSH levels lower than 0.10 mIU/L had a higher risk of developing dementia and cognitive impairment in comparison to those with overt hyperthyroidism.^[17]^ However, mild TSH reductions did not show a significant association with dementia risk. In contrast, a 5-year prospective follow-up study reported that thyroid function was not associated with cognitive function, daily living abilities, or depressive symptoms.^[18]^ These findings have yet to provide a unified consensus on whether AD patients have an increased risk of developing thyroid-related disorders.

The etiology of TH exhibits variations, with discrepancies in thyroid hormone levels among patients during the course of the disease. Depending on the specific thyroid function manifestations, patients may present with normal, hyperactive, or hypoactive thyroid states. At times, throughout the entire disease progression, occurrences of all 3 functional abnormalities are plausible. Moreover, under the influence of the disorder, some patients might progress towards a state of permanent hypothyroidism.^[19]^ Contemporary research suggests that thyroid hormones can bind to thyroid hormone nuclear receptors in brain vascular endothelial cells, promoting the activation of the phosphoinositide 3-kinase (PI3K)/protein kinase B (Akt) signaling pathway. This PI3K/Akt pathway can suppress the expression of glycogen synthase kinase-3 (GSK-3), reduce γ-secretase activity, and hinder the synthesis of amyloid β-protein (Aβ) in the hippocampus. This process subsequently slows down Tau protein phosphorylation, maintaining mitochondria defensive capacity against harmful substances, and alleviating neurotoxic damage.^[20]^ Additionally, thyroid hormones can maintain microtubule stability and flexibility in the axonal distal, reducing microtubule protein dissociation, participating in neuroaxonal nutrient transport, and sustaining axonal transport and neuronal interconnections, thereby preserving normal neural functions such as learning and memory.

However, when thyroid hormone levels in patients become abnormal, the activation of the PI3K/Akt signaling pathway may be inhibited. This can affect the regulation of brain cell conduction systems, promote mitochondrial membrane invagination and increased permeability, leading to the influx of sodium and calcium ions and large molecules into cells, resulting in cytotoxic edema and damage to local neurons. Consequently, this affects neuronal impulse conduction, and promotes phosphorylation of Tau protein, impairing microtubule formation and neuronal interconnections, leading to neuronal loss and degeneration, and ultimately causing cognitive dysfunction.^[21,22]^ Additionally, thyroid hormones can suppress protein kinase A and protein kinase C activities in brain cell membranes and cytoplasm by enhancing the expression of signaling protein genes. Abnormal thyroid hormone expression can lead to the activation of the protein kinase system, which acts as the primary effector of the cell membrane signaling system.^[23,24]^ When the protein kinase system activity is abnormally elevated, it can activate nuclear factor kappa-B (NF-κB), increase the transcription and expression of apoptosis-related genes, initiate neuronal apoptosis, and result in hippocampal neuron loss. This reduces long-term synaptic transmission activity, affecting normal learning behavior and memory functions, leading to cognitive impairment.^[25]^

In the current study at the Mendel Research Institute, single nucleotide polymorphisms (SNPs) are found to be associated with key genes such as PICALM, EPHA1, SORL1, TOMM40, SLC24A4, NECTIN2, CR1, ABCA7, LOC105373605, MS4A6E, CLU, and DSG2. PICALM (phosphatidylinositol-binding clathrin assembly protein) impacts Alzheimer disease (AD) risk by modulating amyloid-β (Aβ) peptide production, transport, and clearance.^[26]^ Ephrin type-A receptor 1 (EPHA1) plays a role in cell and axon guidance as well as synaptic plasticity.^[27,28]^ The sortilin-related receptor 1 (SORL1) gene translates into a scaffolding protein, contributing to amyloid precursor protein (APP) processing.^[29,30]^ Moreover, SORL1 deficiency leads to Aβ peptide accumulation, affecting endosomal degradation and recycling pathways in neurons.^[31,32]^ Complement receptor 1 (CR1) is also a risk gene for sporadic AD, with its locus variations significantly affecting CR1 protein length and the formation of elongated CR1 isoforms implicated in reduced amyloid plaque clearance.^[33]^ ABCA7, a 235 kD protein mainly located in the plasma membrane and Golgi complex, mediates cellular efflux of phospholipids and cholesterol by forming an ATPase-dependent channel, participating in lipid transport and homeostasis.^[34]^ ABCA7 primarily affects phospholipid reverse transport by regulating lipid or cholesterol efflux.^[35]^ MS4A family members play critical roles in different pathological contexts of cancer, infectious diseases, and neurodegenerative disorders.^[36]^ The expression levels of MS4A gene clusters correlate with elevated Braak tangle and Braak plaque scores.^[37]^ CLU influences AD pathogenesis through modulating Aβ aggregation and clearance, neuroinflammation, lipid metabolism, Wnt signaling, copper homeostasis, and regulation of neuronal cell cycles and apoptosis.^[38]^ Desmoglein-2 (DSG2) protein is a transmembrane protein with a broad tissue distribution, encoded by a gene located at 18q12.1, part of the classical cadherin family, facilitating cell-cell adhesion and promoting desmosome assembly. Hence, the SNP alterations in these genes exert a substantial impact on the risk of developing secondary TH among AD patients.^[39,40]^

This study posits a positive correlation between Alzheimer Disease (AD) and increased TH risk. However, intriguingly, the Mendelian randomization analysis conducted with TH as the exposure factor and AD as the outcome in this study did not yield significant results. This observation suggests that while AD may exacerbate the susceptibility conferred by TH, TH itself does not appear to exert a substantial influence on the occurrence of AD. By utilizing genetic variation as a stable and directly measurable exposure factor, the study significantly mitigates confounding influences such as socio-environmental and lifestyle factors, thereby revealing the advantages of Mendelian randomization research. Furthermore, it avoids ethical dilemmas. Nonetheless, the study has several limitations: the 2-sample MR method cannot elucidate non-linear relationships between exposure factors and outcomes; subgroup analyses, including factors such as Intake, stress, and pregnancy, were not conducted, which may result in the presence of differential effect sizes among subgroups; the data originates from European populations, limiting the applicability to other ethnic groups and merely offering reference value. Future studies warrant open access to alternative databases, fostering further investigation. The sample size included in our analysis is relatively limited, which could introduce certain biases into the results.

## Author contributions

**Conceptualization:** Xuejie Song.

**Data curation:** Xuejie Song.

**Funding acquisition:** Xuejie Song.

**Writing – original draft:** Haiyang Yu.

**Writing – review & editing:** Xuejie Song.

## Supplementary Material
